# Impact of the Spittlebug *Mahanarva spectabilis* on Signal Grass

**DOI:** 10.1100/2012/926715

**Published:** 2012-08-13

**Authors:** Tiago Teixeira Resende, Alexander Machado Auad, Marcy das Graças Fonseca, Thiago Henrique dos Santos, Tamiris Moreira Vieira

**Affiliations:** ^1^Federal University of Juiz de Fora, 36036900 Juiz de Fora, Brazil; ^2^Embrapa Dairy Cattle Research Center, 36038-330 Juiz de Fora, Brazil

## Abstract

The aim of this study was to determine the damage in *Brachiaria ruziziensis* (Germain & Edvard) according to the density of and exposure time to adults of *Mahanarva spectabilis* (Distant, 1909) (Hemiptera:Cercopidae). Each plant was kept with 0, 12, 18, or 24 adults of *M. spectabilis* for five or ten days. Then, the insects were removed from the plant, and the following parameters were evaluated: content and loss of chlorophyll, visual damage score, shoot dry mass, and the capability for regrowth. In fact, plants exposed to the highest level of infestation for 10 days showed an 80.97% loss of chlorophyll, which is 25% higher than that shown by the plants exposed for five days. The damage score also increased with infestation levels. In the levels of 12 and 18 adults per plant, the damage score increased with increasing time of exposure. The dry mass content was higher in plants exposed to 24 insects for 10 days, suggesting that the attack of spittlebugs caused premature drying of the plant. These effects caused significant reduction in the number of tillers of infested plants. Our results indicate that exposure to adults of *M. spectabilis* causes significant damage and affects the development and persistence of *B. ruziziensis* plants.

## 1. Introduction

Brazil has distinguished itself in the international milk and beef market because of favorable soil and climatic conditions for cattle rearing; the use of continuously improved cattle breeds adapted to tropical conditions; finally, the solidity of forage introduction, evaluation, and selection programs that have resulted in the release of highly adapted and productive forages [1]. According to the Brazilian Geography and Statistics Institute, in 2011 [2], Brazil had 101 million ha of planted pastures, with the largest commercial cattle herd in the world that feeds mainly on these specially developed forages. However, productivity levels in most Brazilian pastures are considered low because of their state of degradation [3]. In addition, forage production is often further affected by attacks of spittlebugs [4].

The main species of spittlebugs that occur in Brazil are *Notozulia entreriana *(Berg, 1879), *Deois schach* (Fabricius, 1787), and *Deois flavopicta* (Stal, 1854) [4]. Recently, the occurrence of the genus *Mahanarva *has been reported in signal grass, *Brachiaria ruziziensis* [5]. Despite its palatability and forage quality, signal grass is susceptible to spittlebugs [6]. The nymphs of these insects constantly suck the sap of roots, causing yellowing of the plant; adults feed on the shoots, resulting in phytotoxicity [4, 7]. The dry mass yield and forage quality are seriously affected if the attack is severe and extended over time [8, 9], and such attacks have been shown to cause losses ranging from US $840 million to $2.1 billion per year worldwide [10].

Damage to signal grass caused by adult spittlebugs is more severe than the damage caused by nymphs [11–14]. Soon after the adults feed on susceptible signal grass genotypes, chlorotic spots appear in the areas surrounding the feeding points and reduce the chlorophyll content [15]. According to Wang et al. [16], the damage caused by herbivores may reduce the chlorophyll content and negatively affect the photosynthetic capacity of the plants. López et al. [15] measured the loss of chlorophyll in signal grass genotypes attacked by spittlebugs, suggesting that this parameter is an important tool for the indication of damage caused by the attack of these insects.

Most of the studies focused on the selection of forage that showed antibiosis resistance to nymphs of spittlebugs [5, 17, 18]; however, there are some results regarding plant resistance to these insects. However, it is not known the relationship between density and exposure time of plants to *M. spectabilis* adult with the damage caused in signal grass. This knowledge is important to provide recommendations for the control of *M. spectabilis*. Hence, the aim of this study was to determine the damage to *B. ruziziensis* according to the density and exposure time of the plant to adults of *M. spectabilis*.

## 2. Materials and Methods

### 2.1. Plants and Insects

*B. ruziziensis* plants were grown in a substrate mixture (1 L) of soil, sand, and organic fertilizer (3 : 1 : 1) and kept in a greenhouse. To ensure a standardized size and condition of the plants, each shoot was cut 20 cm above ground level 30 days before the start of the experiment and fertilized with 46 mg of urea and 26 mg of potassium chloride, as recommended by soil analysis. On the day of infestation, the plants had an average height of 75.5 ± 1.34 cm, average number of tillers was 8.96 ± 0.45, and average chlorophyll content was 19.91 ± 0.74 SPAD units. 

Nymphs were collected in pastures located in the Embrapa Dairy Cattle Research Station, Brazil, and transferred to vases that contained *B. ruziziensis* plants with exposed roots for feeding. These vases were closed with bags of organza fabric to prevent the nymphs from escaping and were kept in a greenhouse until the emergence of adults, which were then used in the experiment. 

The experiment was conducted in a greenhouse with an average temperature of 27°C and average relative humidity of 81%. These parameters were recorded in a DATALOGGER (HOBOware) and stored for posterior data analyses.

### 2.2. Experiment

Randomized blocks involving four levels of infestation and two exposure times were used in the experiments. Each experimental plot was composed of one *B. ruziziensis* plant kept in a metal cage (70 × 40 × 40 cm) covered with organza fabric. Each plant was kept with 0, 12, 18, or 24 adults of *M. spectabilis*, with the same ratio of males to females. Any dead insects were replaced daily, thus keeping the density of *M. spectabilis* stable for five or ten days. Then, the insects were removed from the plant, and the following parameters were evaluated: content and loss of chlorophyll, visual damage score, shoot dry mass, and the capability for forage regrowth. 

The chlorophyll content was measured in three leaf blades of one plant tiller by using a Minolta SPAD 502 OL chlorophyll meter (Konica Minolta Sensing, Osaka, Japan). Measurements were obtained before infestation (*n* = 12), after five days of infestation (*n* = 12), and 10 days from the initiation of infestation (*n* = 6). For each level of infestation, the average chlorophyll content of the tillers was calculated. Then, the percentage of chlorophyll loss in each treatment group was estimated, as suggested by Deol et al. [19]: percentage chlorophyll loss = [(*U* − *I*)/*U*] × 100, where *U* = SPAD reading for uninfected plants and *I* = SPAD reading for infested plants, at each density level. 

The damage to each plant was assessed visually and assigned a percentage value by three different evaluators, and the average was converted to a damage score of one to five, as proposed by Cardona et al. [20]. After exposure for five (*n* = 6) and ten days (*n* = 6), the plants exposed to different levels of infestation were cut to ground level and their shoots were weighed, thus obtaining the green weight. This material was dried in an oven at 55°C for 72 h to obtain the dry weight. Next, the percentage of dry mass and functional plant loss index (FPLI) proposed by Morgan et al. [21] and modified by Panda and Heinrichs [22] were calculated. This index is calculated on the basis of the damage scores (DSs) and dry weight of uninfested plants (DWUPs) as follows: FPLI (%) = [1 − (DWIP/DWUP) × (1 − DS/5)] × 100, being considered by Smith [23] as a useful tool for quantifying tolerance. 

The vases containing only root systems of signal grass were kept in a greenhouse for 35 days to evaluate the capability for regrowth in plants submitted to different levels of infestation by *M. spectabilis*. Every seven days, the number of tillers was counted, and reduction in the number of tillers (RNT) was calculated as follows: RNT (%) = [(number of tillers of uninfested plant − number of tillers of infested plant)/number of tillers of uninfested plant] ∗ 100.

### 2.3. Statistical Analysis

The average chlorophyll content of each plant, damage scores, green shoot weight, dry weight, and dry mass were evaluated using analysis of variance (ANOVA). Significant values (*P* ≤ 0.05) were subjected to regression analysis regarding infestation levels of *M. spectabilis* or the averages were compared using Tukey test (*P* ≤ 0.05) to evaluate the effect of exposure time of the grass to the spittlebugs (five and ten days).

The emission of tillers, percentage of chlorophyll loss, and functional *B. ruziziensis *loss index were evaluated using ANOVA, and when significant (*P* ≤ 0.05), the mean values were compared using Tukey test (*P* ≤ 0.05).

The analyses were performed using the program SISVAR 5.3 [24] (Federal University of Lavras, MG, Brazil). The correlation between chlorophyll content and the damage scores was evaluated using the Pearson test in the SAEG 9.1 [25] (Federal University of Viçosa, MG, Brazil) program.

## 3. Results and Discussion

**Figure 1:**
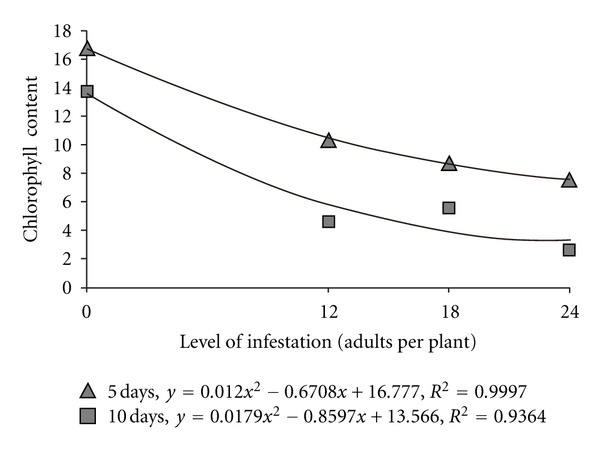
Relationship between infestation levels of adult *M. spectabilis* and chlorophyll content (SPAD unit) in *B. ruziziensis* over 5 or 10 days.

We observed no significant differences in the chlorophyll content of plants before exposure to *M. spectabilis* adults, regardless of the infestation level that would later be introduced (*F* = 0.61; *P* = 0.60); this confirmed the standardization of the plants submitted to treatments. On subsequent evaluations, the content of chlorophyll decreased significantly with increasing infestation levels of *M. spectabilis *on plants exposed for either five days (*F* = 7.10; *P* < 0.01) or ten days (*F* = 22.05; *P* < 0.01), and quadratic regressive curves were obtained for the two exposure times (Figure 1).

**Figure 2:**
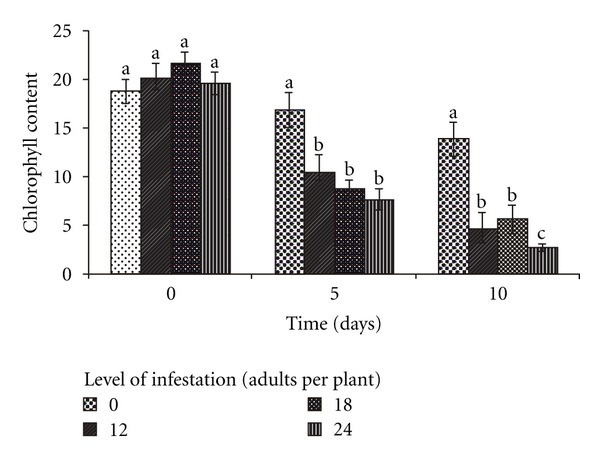
Relationship between chlorophyll content (SPAD unit) of *B. ruziziensis* and exposure time (0, 5, and 10 days) at different infestation levels of adult *M. spectabilis*. Mean values followed by the same letter within the levels of infestation did not differ by Tukey test.

Compared to the initial measurement, a significant reduction in chlorophyll content was observed in the plants exposed to 12 (*F* = 14.77; *P* < 0.01) and 18 adults (*F* = 23.06; *P* < 0.01) during five and ten days. When exposed to 24 insects, there was a significant difference among the three exposure times (*F* = 53.14; *P* < 0.01); when the plants were exposed to insects for 10 days, the chlorophyll content was 2.8-fold less than that observed when they were exposed for five days. It is important to highlight that the experimental period alone did not cause the natural reduction in the chlorophyll content, since the content in the uninfested plants did not differ significantly with time (*F* = 2.04; *P* = 0.15) (Figure 2). The relationship of both exposure time and infestation level with chlorophyll content has been reported in other insects by Deol et al. [26] and Diaz-Montano et al. [27], who found that increase in the number of aphids and exposure time reduced the chlorophyll content of wheat and soybean plants, respectively. In this study, we observed that exposure of signal grass to 12 adults of *M. spectabilis* for five days was sufficient to cause significant reduction in the chlorophyll content.

Plants exposed to higher levels of infestation over 10 days lost 80.97% of their chlorophyll; a loss that was 25% greater than that in the plants exposed for 5 days (*F* = 11.41; *P* < 0.001). In contrast, no significant difference was found for chlorophyll loss among the exposure times at levels of 12 (*F* = 4.19; *P* = 0.06) and 18 (*F* = 0.54; *P* = 0.47) insects; however, compared to uninfested plants, these losses were greater than 40%. These results confirmed those obtained by López et al. [15] who identified chlorophyll loss in signal grass genotypes infested with adults of two other spittlebug species, *Aeneolamia varia* and *Zulia carbonaria*. This reduction has also been reported in wheat plants infested with *Schizaphis graminis* [26] and soybean plants infested with *Aphis glycines* [27].

The chlorophyll loss in *B. ruziziensis* infested with adults of *M. spectabilis* may affect the photosynthetic capability of the plant. The toxic saliva injected by the adults that feed on the shoots of the grass interferes with photosynthetic activity [28]. Moreover, according to Nabity et al. [29], when the insects feed on the xylem or phloem, water transport, stomatal aperture, and sucrose transport are affected, thereby reducing photosynthesis in the remaining leaf tissue of the attacked plants. Reduction in chlorophyll content and consequent reduction in photosynthetic capability have been observed for other sucking insects [30, 31]. According to Welter [32], 50% of the studies that examined insect-plant interactions have reported a loss of photosynthetic capability in the plants. 

**Figure 3:**
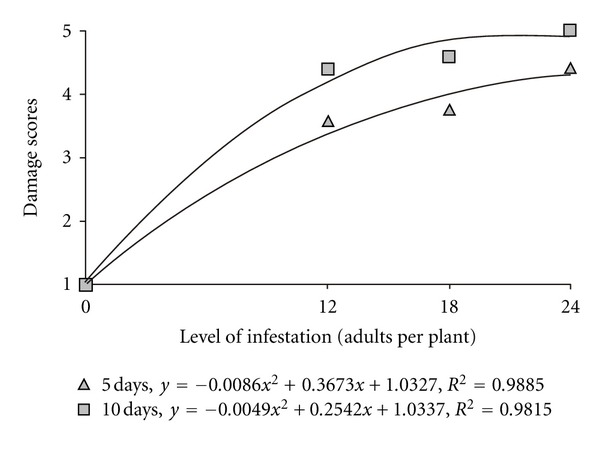
Relationship between infestation levels of *M. spectabilis* adults and damage scores for *B. ruziziensis* over 5 or 10 days.

We observed that the damage scores increased with increasing levels of infestation of *M. spectabilis* in the plants exposed to adults over five days (*F* = 84.59; *P* < 0.0001) or ten days (*F* = 114.11; *P* < 0.0001). Coefficients of determination (*R* ^²^) were highly significant, indicating good adjustment of curves to the data obtained (Figure 3). Moreover, it was found that the damage in the plants was higher with increase in the exposure time at levels of infestation of 12 (*F* = 5.77; *P* < 0.05) and 18 adults (*F* = 11.08; *P* < 0.01). However at the higher level of infestation no significant difference was found between the times of exposure (*F* = 2.60; *P* = 0.12), demonstrating that a five-day period was sufficient to cause damage near the maximum end of the scale used.

It is important to highlight that, even at lower insect density and shorter exposure time, the damage score was still significant (3.5). Similar values were reported by Cardona et al. [18, 20] when they used *B. ruziziensis*, *B. decumbens*, and hybrids of this forage. According to López et al. [15], these damages seem irreversible because even 10 days after the removal of adults of *A. varia* and *Z. carbonaria*, none of the evaluated genotypes of *Brachiaria* showed any signal of recovery of leaves.

Cardona et al. [18] observed that most of the hybrids resistant to nymphs are susceptible to adults of spittlebug, suggesting that programs to improve forage should select plants that are resistant to attack by adults. The capability of *M. spectabilis* adults to cause significant damage to plants was observed in this study (Figure 3), thus confirming the need for improvement programs to include adults in future tests.

Correlation analysis showed that damage scores were inversely related to the chlorophyll content of signal grass after both five (*T* = −3.02; *P = *0.002) and ten (*T* = −3.13; *P = *0.002) days of exposure of the plants to *M. spectabilis*. López et al. [15] also reported high correlation between the damage scores and the percentage of chlorophyll loss in genotypes of signal grass infested with adult spittlebugs.

**Figure 4:**
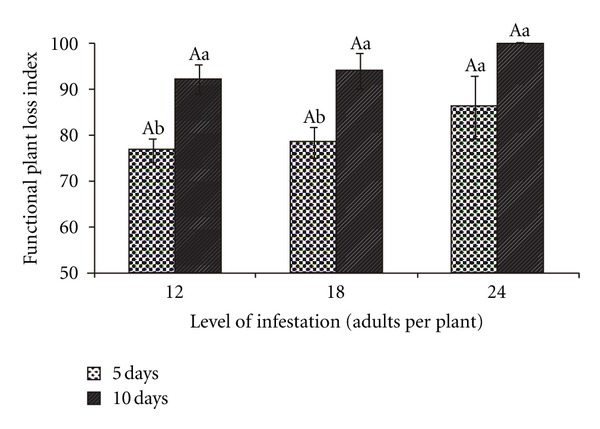
Functional plant loss indexes (%) after 5 or 10 days of exposure to 3 levels of infestation by *M. spectabilis* adults. Bars with the same lowercase letters within the level of infestation and bars with the same capital letters between the levels of infestation did not differ by Tukey test.

We observed that even the lowest density of *M. spectabilis* was enough to cause a functional loss greater than 75% in *B. ruziziensis*. The losses were significantly greater when the plants were exposed to densities of 12 (*F* = 14.54; *P* < 0.001) and 18 (*F* = 9.07; *P* = 0.01) insects for longer exposure times. At a density of 24 insects, there was no significant difference in functional loss between the exposure times (*F* = 2.92; *P* = 0.11), and shorter exposure time was enough to cause a loss greater than 86% (Figure 4). López et al. [15] showed a functional loss index of 93.4% and 100% when signal grass was infested with *A. varia* and *Z. carbonaria*, respectively. Cardona et al. [18] observed a functional loss index of 87.6% in *B. decumbens* attacked by five *Z. carbonaria *adults after 10 days of exposure. The above results show that, independent of the species of adult spittlebug, there is a similarity in functional loss in *Brachiaria*. The functional loss index measures the tolerance of plants to insects (Morgan et al. [21], modified by Panda and Heinrichs [22]), and according to López et al. [15], it is the best index to estimate the tolerance of signal grass to spittlebug. The values obtained in our study show that *B. ruziziensis *does not tolerate the attack of 12 adults of *M. spectabilis *per plant after five days; therefore, the levels of infestation of this cercopid should be kept under this density.

No significant changes in the green weight of the plants were observed at the different levels of infestation after five days of exposure to the insect (*F* = 1.14; *P* = 0.35). However, 10 days of exposure resulted in reduction in the green weight (*F* = 3.03; *P* = 0.05), with the variables being correlated in a quadratic fashion (*y* = 0.0101*x* ^²^ − 0.6696*x* + 20.283; *R* ^²^ = 0.9719). The increase in density of adults of *M. spectabilis* did not result in significant changes in either dry weight or the percentage of dry mass of infested signal grass, regardless of the exposure time.

Compared to the plants exposed for only five days, those exposed for ten days to 24 *M. spectabilis* adults showed a significant increase in dry mass content (*F* = 6.27, *P* = 0.01). Valério and Nakano [12] also reported an increase in dry mass content in *B. decumbens *infested with high densities of adults of *Zulia entrerriana*. Weaver and Hibbs [33] and Marthus and Pienkowski [34] who studied *Philaenus spumarius* in alfalfa and red clover, respectively, and Fagan [35], who studied *Prosapia bicincta* in *Digitaria decumbens*, also observed increases in the percentage of dry mass content in these host plants as a result of damage caused by spittlebugs. The increase in dry mass found in the above results is a negative effect, since damages imposed by the spittlebug result in early drying of the plants. This reduces the green weight and consequently increases the dry mass, which is the result of the dry weight divided by the green weight. Moreover, the attack of spittlebugs reduces the palatability of grasses, specifically reducing the acceptance of the forage by grazing animals such as cattle [12].

**Figure 5:**
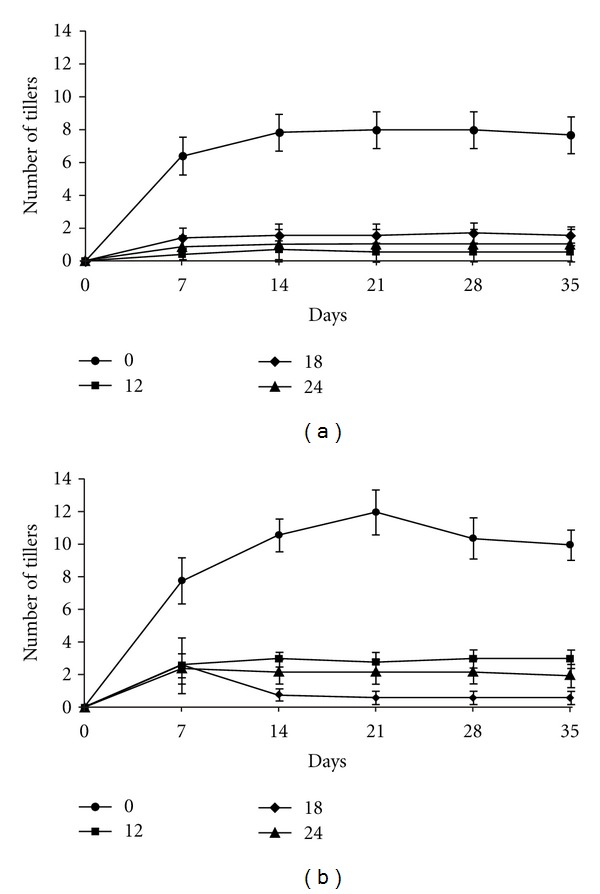
Number of tillers after the *B. ruziziensis* plants were cut and subjected to different levels of infestation by *M. spectabilis* adults over 5 (a) or 10 (b) days.

When regrowth was evaluated after cutting the plants, significant difference in the number of tillers of plants infested at densities of 12, 18, and 24 adults in both exposure periods was observed (*P* < 0.0001). In the first evaluation 7 days after cutting, the infested plants in the five- and ten-day exposure groups showed an average of 0.89 and 2.53 tillers, respectively. After five and ten days of exposure, the uninfested plants showed an average of 6.42 and 7.8 tillers, respectively. In the following evaluations, the number of tillers was maintained for plants exposed to insects for five or ten days (Figures 5(a) and 5(b)).

The reduction in the number of tillers ranged from 67% to 90% over the three levels of infestation, regardless of the exposure time to the insect. According to Valério [36], frequent attacks of spittlebug, combined with other factors, may reduce root-system volume and, thus, reduce the persistence of the grass. Our study supports this hypothesis, since we observed a reduction in the number of tillers in plants infested by *M. spectabilis*.

According to López et al. [15], after adult spittlebugs feed, chlorotic spots appear in the area around the feeding points and progress to form white or yellow stripes from the tip to the base of the leaf blade. In our study, the damages caused by *M. spectabilis* to the shoots were reflected in its root system and resulted in reduced regrowth. Valério [36] reported that plants of *B. decumbens*, susceptible to antibiosis by spittlebugs, did not exhibit regrowth after infestation with spittlebugs of the genus *Mahanarva*. In more severe attacks, even the “Marandu” cultivar resistant to antibiosis by spittlebug nymphs showed only a small amount of recovery [36].

The lowest density of *M. spectabilis *adults and the shortest exposure time, 12 adults over five days, were sufficient to cause damage and affect the development and persistence of *B. ruziziensis* plants, highlighting that the impact caused by adult spittlebugs in signal grass is very severe.
